# Deaths of cyclists in london: trends from 1992 to 2006

**DOI:** 10.1186/1471-2458-10-699

**Published:** 2010-11-15

**Authors:** Andrei S Morgan, Helen B Dale, William E Lee, Phil J Edwards

**Affiliations:** 1 Department of Epidemiology and Public Health, London School of Hygiene and Tropical Medicine, Keppel Street, London WC1E 7HT, UK; 2 College of Medical and Dental Sciences, University of Birmingham, Edgbaston, Birmingham, B15 2TT, UK; 3 Institute of Psychiatry, King's College London, De Crespigny Park, London, SE5 8AF, UK

## Abstract

**Background:**

Cycling is an increasingly important mode of transport for environmental and health reasons. Cycling fatalities in London were previously investigated in 1994 using routinely collected data. Since then, there have been shifts in the modes of transport used, and in transport policies. We sought to replicate the previous work using data on cyclist deaths in London between 1992 and 2006, specifically investigating whether heavy goods vehicles continued to pose a threat.

**Methods:**

Observational study based on analysis of time series of police road casualties data, 1992 to 2006, in London, UK. The main outcome measures were cyclists killed in road traffic collisions. Poisson regression and chi-squared test for homogeneity were used to assess time effects. Travel flow data was then used to estimate annual fatality rates per 100,000 cyclists per kilometre.

**Results:**

From 1992 to 2006 there was a mean of 16 cycling fatalities per year (range 8-21). 146 deaths (60%) were in inner London and 96 in outer London. There was no evidence for a decline over time (p = 0.7) other than a pronounced dip in 2004 when there were 8 fatalities. Freight vehicles were involved in 103 of 242 (43%) of all incidents and the vehicle was making a left turn in over half of these (53%). The fatality rate ranged from 20.5 deaths in 1992 to 11.1 deaths in 2006 per 100,000 estimated cyclists per kilometre (rate ratio 0.54, 95% confidence interval 0.28 to 1.03).

**Conclusions:**

There is little evidence fatality rates have fallen. Freight vehicles over 3.5 tonnes continue to present a disproportionate threat; they should be removed from urban roads and more appropriate means of delivery of essential goods found.

## Background

Cycling is an increasingly important mode of transport for environmental and health reasons [1]. There is no "carbon waste" and it is protective against obesity and related conditions [2]. Deaths of cyclists in London were previously investigated using data from 1985 to 1992 [3] looking at the characteristics of the cyclists and vehicles in the collision. The results showed adults were more frequently involved in fatal crashes than children, and heavy goods vehicles were over-represented, leading them to conclude:

"In inner London, in relation to their traffic volume, heavy goods vehicles are estimated to cause 30 times as many cyclists' deaths as cars and five times as many as buses. Until the factors leading to this excess risk are understood, a ban on heavy goods vehicles in urban areas should be considered."

Since 1994, there have been changes to London's cycling and other transport policies. In particular there has been a shift in the modes of transport utilised [4]. In 2006, transport policy in London changed to include a target of a 50% reduction by 2010 in cyclists killed and seriously injured in road traffic incidents compared with the period 1994-1998 [5]. This is to complement a European Union initiative to halve road traffic deaths by 2010 [6]. We aimed to replicate the previous study by assessing trends in fatalities of cyclists in London between 1992 and 2006 and, specifically, investigating whether heavy goods vehicles continued to pose a threat to cylists.

## Methods

### Data sources

STATS19 data are legally required to be collected by police following a road traffic collision involving an injury. Reporting may occur following police attendance at the scene of the incident, or subsequently by an involved individual at a police station. We obtained a file containing a list of all collisions recorded in the STATS19 dataset held by Transport for London (TfL; http://www.tfl.gov.uk) from the beginning of January 1992 to the end of 2006 - 15 years in total. This range included an overlap with the previous study [3] to allow for verification and a period after the introduction of the congestion charge. The dataset we used is available on request from TfL. Additionally, TfL supplied us with baseline data about fatalities for 2001-2006 with which to corroborate numbers. These data have since been published in a report containing total annual casualties from 1986 to 2007 [7]. Due to the possibility of less severe crashes being under-reported in the database [8], we restricted our analysis to fatal collisions.

Data on cycle usage in London was acquired from the London Travel Report 2007 [7], produced by TfL. This report compiles data from a number of sources, including the National Road Traffic Survey, organised by the Department for Transport, and the London Travel Demand Survey (LTDS). It also includes data from TfL's own automated traffic counters. Travel flow data for major roads, in which the estimated total vehicle distance travelled is divided by the length of roads included, was available for cycling for the time period of the present study. Equivalent data for motorised vehicles was available from 1993-2006. The data on cycle flows is publicly available and was downloaded from http://www.tfl.gov.uk/travelinlondon in September, 2009.

Ethical approval for this study was granted by the Research and Ethics Committee of the London School of Hygiene and Tropical Medicine.

### Data analysis

Casualty age and sex were examined, as well as any additional information about the collision that was available. General features of the incident noted were specific location details, including the class of road, weather, and lighting conditions. A brief free-text description of each incident was also available.

The data were restricted to only those collisions occurring within the boundaries of greater London. Numbers of fatalities per year were compared by sex, age group and incident location (inner - City of London and 12 boroughs - or outer London - 20 boroughs); we also investigated any difference in fatalities in relation to the introduction of the central London congestion charge on 14 February 2003. Vehicles involved in incidents were categorised as cars (including taxis and light goods vehicles with a maximum laden weight of less than 3.5 tonnes), freight vehicles - which incorporated medium (3.5-7 tonnes) and heavy (> 7 tonnes) goods vehicles (HGVs) - and other (public service vehicles, powered two wheel vehicles, and other bicycles). Evidence for differences in the distributions of events by casualty characteristics, place of collision and time was assessed using the chi-squared test. Poisson regression was used to estimate change over time in the numbers of events, adjusting for changes in exposure (measured using travel flow data on numbers of cyclists per year). We calculated annual fatality rates per 100,000 cyclists per kilometre and estimated a rate ratio with a 95% confidence interval for the overall change during the study period.

All analyses were conducted using STATA version 10 (STATACorp, USA).

## Results

247 collisions were identified in London between 1^st ^January 1992 and 31^st ^December 2006 which involved cyclists and at least one fatality. Two collisions involved the death of the driver of another vehicle and three involved only the deaths of pedestrians; these were excluded, leaving 242 collisions with a cyclist fatality. Data were missing on casualty age in 10 collisions; additionally, descriptive information about the crashes was missing for collisions during 2005-06.

There was a mean of 16.1 cycling fatalities per year (range 8-21). This remained relatively constant (p = 0.70) other than a pronounced dip in 2004 when there were only 8 fatalities. 146 deaths (60%) were in inner London and 96 in outer London; again, this was relatively consistent throughout the study period (p = 0.74).

Almost three quarters of fatalities were male (n = 177), and there was no evidence of a difference over the time period studied. There was a difference in the age distribution by sex, though, with the majority of deaths being male in both younger and older age groups, and only the 18-24 age group involving more female deaths (12, or 54.6%, vs 10 male deaths).

There were more deaths of female cyclists in inner London than outer London (48 and 17 deaths, respectively) compared with men (98 and 79 fatalities; p = 0.009). Deaths of women were more common during daylight (91% of females as opposed to 74% of males; p = 0.004). There was no difference between the sexes for the type of road on which the incident occurred. Approximately three quarters of the incidents (n = 184) overall occurred on, or at a junction with, a major road.

Of the 232 deaths where age was available, 28 (12%) were children aged 16 years or under. Six percent (8 of 137) of fatalities in inner London were aged 16 or under compared with 21% (20 of 95) in outer London (p < 0.001). There were no important differences between children and adults in the frequency of fatalities occurring in bad weather or when it was dark.

For all these results, complete data are shown in additional file 1.

Freight vehicles were involved in over 40% (103 of 242) of all the incidents in the study period and, of these, the vehicle was making a left turn in over half (53%). Other manoeuvres reported included turning right, going ahead, and overtaking. Cars, light goods vehicles and taxis combined were involved in a similar number of incidents to freight vehicles (105 of 242). Buses, motor cycles, other pedal cycles or an unknown other vehicle were involved in 12, 6, 2 and 9 incidents respectively. The total number of vehicles involved (other than the cyclist) was 251, with 12 collisions involving more than one vehicle other than the cyclist fatality, and 5 (2%) incidents involving the cyclist alone without any motor vehicle being involved. Full data are available in additional file 2. To put these figures into context, for the period in which traffic flow data is available (1993-2006), goods vehicles (as defined by TfL) accounted for approximately 4% of traffic flow, cars and light vans 91%, and buses and powered two-wheelers 2% each [7].

Comparing other vehicle type with casualty sex showed that there were fewer women than men involved in incidents with cars (13 vs 84 deaths) than with another vehicle (52 vs 93 deaths; p < 0.001). More men (56 fatalities) were killed in an incident with a freight vehicle than women (47 deaths; p < 0.001). There was no difference between the sexes for fatalities caused by freight vehicles turning left (p = 0.35).

Fewer than 20% of incidents happened either on the boundary of or in the central London Congestion Charging Zone for the time periods before and after the introduction of the congestion charge, and there was no significant difference between these periods (p = 0.67). However, the number of fatalities occurring within the congestion charging area was small: 15 of 175 collisions in the 12 year period before the introduction of the charge, and 7 of 67 incidents following the introduction (additional file 3).

### Death rates over time

Travel flow data showed an important increase in the number of journeys made by bicycle in London. During the study period of 1992-2006, estimated traffic flows for cycling on major roads in London increased from 240 to 470 bicycles per kilometre per day [7], an increase of 95.8%. Overall transport flows on major roads increased by only 4.4% between 1993 and 2006 - the dates for which full data were available [7]. However, in relation to motorised vehicles, cycling flows increased during this time by 73.2%, from 0.85% to 1.48% of the total traffic flows on major roads. The fatality rate fell from 20.5 deaths in 1992 to 11.1 deaths in 2006 per 100,000 estimated cyclists per kilometre, equivalent to an overall decrease of 46% (rate ratio 0.54, 95% confidence interval 0.28 to 1.03). Data are shown in additional file 4.

## Discussion

### Principal findings

Despite evidence for increases in the amount of cycling in London over the last 15 years, the number of cyclists who have been killed has remained constant. The biggest threat remains freight vehicles, involved in more than 4 out of 10 incidents, with over half turning left at the time of the crash. We did not find any difference in the number of fatalities in central London following the introduction of the congestion charge on the 14th February, 2003, although the majority of deaths overall occurred in inner London.

### Strengths and weaknesses of this study

This builds on the study by Gilbert and McCarthy [3] in 1994 - by including estimates of fatality rates. However, as previously, the number of cyclists killed in London remains small, meaning that even if trends were present, they may not have been detected. Our estimates of fatality rates are likely to be over-estimates, as the denominator was based on data from TfL's London Travel Report 2007 which took data from the National Road Traffic Survey, whereas the death counts included all roads. Automated cyclist counters have since been placed on major roads in London, although these currently cover less than 5% of the road network. Nevertheless, the same types of data were used consistently for our calculations, thus allowing for the possibility of a trend being detected. Additionally, this is the first study of cycling deaths of which we are aware to include fatality rates. Fatality rates are difficult to obtain as there is no estimate of the miles cycled per year. We have compromised by providing the fatality rates per 100,000 estimated cyclists per kilometre on the roads of London. However, for an individual cyclist, it would be more useful to know the fatality rate per million miles cycled - a figure that currently cannot be obtained.

### Relationship to other studies

This is the first study since Gilbert and McCarthy [3] to examine cycling fatalities in a single, major urban area. Other studies have analysed data on a regional basis, including both rural and urban environments [9], compared differences in cycling safety between different populations [10,11], or compared differences between different modes of transport [12]. Transport for London have also published data on the numbers killed and seriously injured (KSI) in the London area taken from the same dataset (STATS19) [5] but this report did not perform any statistical analysis. Furthermore, the figures for non-fatal casualties are likely to be subject to greater reporting bias, with a recent analysis performed on behalf of Transport for London indicating that only about 65-70% of all casualties are reported to the police - and, therefore, represented in the STATS19 dataset [8]. Furthermore, the data in STATS19 appears to underestimate the severity of injury, such that only about two thirds of serious injuries were classified as such.

Our findings in relation to freight vehicles are consistent with the previous study's findings and are more worrying as we have used a stricter definition of 'freight' vehicle than the heavy goods vehicle (HGV) classification that was used before (incorporating vehicles over 1.524 tonnes unladen weight) [3]. We also concur with the conclusion that freight vehicles are disproportionally involved in collisions fatal to cyclists: using the data from our study, freight vehicles are approximately 24 times more likely to be involved in a fatal incident than cars, 4 times as likely as buses and 8.5 times as likely as motorcycles. We did not replicate, however, the findings of previous studies which have suggested that increased levels of cycling lead to decreased fatality rates [13]. This may be because there are many different factors influencing cycling fatalities and, in particular, that the circumstances in a heavily urbanised environment such as London are markedly different from the mixed urban, suburban and rural environments that featured in the other study. Our study may also have been underpowered to detect an effect. This was suggested by the extended trend analysis of deaths alone (from 1986 to 2007). Due to lack of data and differences in collection methodologies throughout the study period, however, our estimates are the best available.

The findings of this study with respect to freight vehicles are consistent with a report produced by the London Road Safety Unit which combined data from STATS19, police collision investigation files and enhanced vehicle registration data from the Department for Transport. This report - which has never been published but is in the public domain - found over half of the fatalities involved a left-turning freight vehicle, and that "lack of experience of drivers or riders does not appear to be a problem." The problem of [left] turning goods vehicles has been analysed in depth in another paper [14], which highlights that the principal contributory factor is the limited field of vision from the driver's cab of nearby road objects. This is because freight vehicles are not designed for urban environments such as London, instead being intended for transporting goods on large, suburban roads and motorways with free-flowing traffic and few vulnerable road users.

### Policy implications and future research

The safety of cyclists is paramount for cities such as London that are beginning to promote cycling as a means of mass transport. Our study identifies areas for improvement in safety by highlighting the dangers that continue to be posed by HGVs. In London, the recent consultation in relation to the development of the Mayor of London's Transport Strategy contains 9 out of 129 proposals directly related to cycling [15]. One relates to cycling safety in general and one relates to cycling safety in relation to HGVs (enhancing the use of sideguards on HGVs, as well as trying to increase the number of freight vehicles with "electronic warning devices that detect cyclists").

Our study also found that the majority of fatalities were adults and males - probably reflecting to a certain degree the people who cycle. However, this is hard to know as there is no good data on the type of people who cycle, nor how often and how far. Even if these data were available, it may be likely to reflect people who use their bikes little and often (e.g. for the daily commute) rather than those who take longer but less frequent rides, although this is currently unknown. A further area for investigation is the impact of high visibility clothing and cycle helmets on fatality rates. At present, there are mixed opinions on the benefits of cycle helmets [16,17], and there were no studies found in a Cochrane Review looking at the impact of high visibility aids in cyclist-motor vehicle collisions [18].

## Conclusion

To conclude, as in the study by Gilbert and McCarthy [3], our principal finding reflects the danger of motorised vehicles to cyclists and, in particular, the danger associated with freight vehicles. To prevent cyclists being killed by HGVs, large freight vehicles (> 3.5 tonnes maximum laden weight) should be removed from urban roads and more appropriate means of delivery of essential goods found. River or rail transport could bring goods into urban environments, for example, and light goods vehicles could then be used for local re-distribution.

## Competing interests

This study is based solely on work by the authors. Transport for London provided the data but did not have any influence on the study design, analysis or production of the final report. All four of the authors are keen cyclists; additionally, AM, HD and WL founded the Bicycle Users' Group at the London School of Hygiene and Tropical Medicine, and AM has been co-Chair since 2007. None of the authors received any financial payments in relation to this study or to their interest in cycling. WL is supported by the Medical Research Council.

## Authors' contributions

AM, HD and WL conceived the idea and PE sourced the data; all authors contributed towards the planning and design of the study. Analysis was conducted by AM, HD and WL. The paper was initially drafted by AM and all authors contributed to the final version. AM is the guarantor of the article.

## Pre-publication history

The pre-publication history for this paper can be accessed here:

http://www.biomedcentral.com/1471-2458/10/699/prepub

## Supplementary Material

Additional file 1 **Cycling fatalities in London by year, 1992 - 2006**. Click here for file

Additional file 2 **Vehicle information for collisions involving a cyclist fatality in London, 1992 - 2006**. Click here for file

Additional file 3 **Cyclist fatalities before and after the introduction of the Central London Congestion Charging Zone (CCZ)**. Click here for file

Additional file 4 **Traffic flows and corresponding estimated cycling fatality rates, 1992 - 2006**. Click here for file
