# Supplementary material for: Deaths of cyclists in london: trends from 1992 to 2006
Source: BMC Public Health. 2010 Nov 15;10:699. doi: 10.1186/1471-2458-10-699 (PMC2992064; doi:10.1186/1471-2458-10-699)
Supplement: Additional file 1 — Cycling fatalities in London by year, 1992 - 2006. [file 1471-2458-10-699-S1.PDF]

Additional file 1: Cycling fatalities in London by year, 1992 – 2006

Category		Year															Total
		1992	1993	1994	1995	1996	1997	1998	1999	2000	2001	2002	2003	2004	2005	2006	
Sex of casualty [1]																	
	Male	14	10	11	11	17	10	6	8	11	17	16	12	6	17	11	177
	Female	4	8	4	4	3	2	6	2	3	4	4	7	2	4	8	65
Age group																	
	0 – 9	0	0	1	0	0	0	1	0	0	1	3	1	0	0	0	7
	10 – 17	3	3	0	2	2	2	1	1	1	4	1	2	1	0	0	23
	18 – 24	1	1	0	1	4	0	3	1	1	2	1	2	1	4	0	22
	25 – 29	1	3	1	2	4	1	1	1	2	2	3	3	1	3	4	32
	30 – 29	6	4	3	3	3	6	1	3	2	1	5	3	3	6	6	55
	40 – 49	1	1	2	3	2	2	3	1	2	1	3	4	1	1	3	30
	50 – 64	4	3	3	2	1	1	1	2	1	2	1	2	0	5	4	32
	65 – 79	1	3	4	1	2	0	0	1	2	6	2	0	1	0	1	24
	80 +	1	0	0	0	1	0	0	0	0	1	0	1	0	2	1	7
	Missing	0	0	1	1	1	0	1	0	3	1	1	1	0	0	0	10
Region [2]																	
	Inner London	8	10	8	8	10	8	7	7	9	10	13	15	5	15	13	146
	Outer London	10	8	7	7	10	4	5	3	5	11	7	4	3	6	6	96
Weather conditions																	
	Fine	15	18	14	14	19	12	12	9	13	20	16	18	7	18	18	223
	Raining	2	0	1	1	1	0	0	1	0	1	2	1	1	2	1	14
	Other/unknown	1	0	0	0	0	0	0	0	1	0	2	0	0	1	0	5
Lighting conditions																	
	Light	17	13	13	12	13	10	11	10	8	16	17	12	6	17	14	189
	Dark	1	5	2	3	7	2	1	0	6	5	3	7	2	4	5	53
Total		18	18	15	15	20	12	12	10	14	21	20	19	8	21	19	242

1. Chi-squared test for homogeneity, p=0.463
2. Chi-squared test for homogeneity, p=0.744
