# Supplementary material for: Deaths of cyclists in london: trends from 1992 to 2006
Source: BMC Public Health. 2010 Nov 15;10:699. doi: 10.1186/1471-2458-10-699 (PMC2992064; doi:10.1186/1471-2458-10-699)
Supplement: Additional file 2 — Vehicle information for collisions involving a cyclist fatality in London, 1992 - 2006. [file 1471-2458-10-699-S2.PDF]

Additional file 2: Vehicle information for collisions involving a cyclist fatality in London, 1992 – 2006

Category	Year															Total
	1992	1993	1994	1995	1996	1997	1998	1999	2000	2001	2002	2003	2004	2005	2006	
Total number of vehicles involved per accident[1]																
1	0	0	1	0	2	0	0	0	0	0	1	0	0	1	0	5
2	16	18	13	14	18	11	11	10	14	20	18	17	8	20	17	225
3+	2	0	1	1	0	1	1	0	0	1	1	2	0	0	2	12
Type of 'other' vehicles involved[2]																
Bicycle	2	0	0	0	0	0	0	0	0	0	0	0	0	0	0	2
PTW[3]	0	1	0	0	0	0	0	0	0	2	0	1	0	1	1	6
Car[4]	9	8	8	11	9	3	4	3	5	8	9	12	2	11	15	117
Freight vehicle[5]	8	9	5	2	5	7	9	7	8	8	11	8	4	6	6	103
Bus[6]	1	0	1	0	3	1	0	0	0	3	0	1	2	2	0	14
Other	0	0	1	3	1	2	0	0	1	1	0	0	0	0	0	9
Total number of 'other' vehicles involved[3]	20	18	15	16	18	13	13	10	14	22	20	22	8	20	22	251
Number of accidents involving...																
Bicycles	2	0	0	0	0	0	0	0	0	0	0	0	0	0	0	2
PTW[3]	0	1	0	0	0	0	0	0	0	2	0	1	0	1	1	6
Cars[4]	9	8	7	8	9	3	3	3	5	7	8	9	2	11	13	105
Freight vehicles[5]	8	9	5	2	5	7	9	7	8	8	11	8	4	6	6	103
Bus[6]	1	0	0	0	3	0	0	0	0	3	0	1	2	2	0	12
Other	0	0	1	3	1	1	0	0	1	1	0	0	0	0	0	8

1. Includes the cyclist who died. 2. Excludes the cyclist who died. 3. PTW = powered two-wheeler (mopeds and motorcycles). 4. 'Car' includes taxis and light goods vehicles (<3.5 tonnes unburden weight). 5. 'Freight vehicle' includes goods vehicles of 3.5 tonnes unburden weight or greater. 6. 'Bus' includes buses and coaches.
