# Supplementary material for: Deaths of cyclists in london: trends from 1992 to 2006
Source: BMC Public Health. 2010 Nov 15;10:699. doi: 10.1186/1471-2458-10-699 (PMC2992064; doi:10.1186/1471-2458-10-699)
Supplement: Additional file 3 — Cyclist fatalities before and after the introduction of the Central London Congestion Charging Zone (CCZ). [file 1471-2458-10-699-S3.PDF]

Additional file 3: Cyclist fatalities before and after the introduction of the Central London Congestion Charging Zone (CCZ)[1].

Time period	Inside[2]	On the boundary[2]	Outside[2]	Total
Pre-congestion charge	15 (8.6%)	11 (6.3%)	149 (85.1%)	175
Post-congestion charge	7 (10.4%)	6 (9.0%)	54 (80.6%)	67

1. The CCZ was introduced on 14th February, 2003. P = 0.67 (Chi-squared test for homogeneity).
2. Fatalities were divided into those occurring inside, on the boundary with, and outside the CCZ.
