# Supplementary material for: Deaths of cyclists in london: trends from 1992 to 2006
Source: BMC Public Health. 2010 Nov 15;10:699. doi: 10.1186/1471-2458-10-699 (PMC2992064; doi:10.1186/1471-2458-10-699)
Supplement: Additional file 4 — Traffic flows and corresponding estimated cycling fatality rates, 1992 - 2006. [file 1471-2458-10-699-S4.PDF]

Additional file 4: Traffic flows and corresponding estimated cycling fatality rates, 1992 – 2006

Year	Number of fatalities	Traffic flow [1000 vehicles/kilometre/day[1] (%)]			Estimated number of cyclists per kilometre per year	Estimated death rates per 100,000 cyclists per kilometre per year[2]
		Motor vehicles	Pedal cycles	Total		
1992	18	-- (--)	0.24 (--)	-	87660	20.5
1993	18	30.3 (99.15)	0.26 (0.85)	30.52	94965	19.0
1994	15	30.8 (99.13)	0.27 (0.87)	31.09	98618	15.2
1995	15	30.8 (99.16)	0.26 (0.84)	31.09	94965	15.8
1996	20	31.1 (99.08)	0.29 (0.92)	31.40	105922	18.9
1997	12	31.3 (99.11)	0.28 (0.89)	31.58	102270	11.7
1998	12	31.3 (99.18)	0.26 (0.82)	31.60	94965	12.6
1999	10	31.9 (99.19)	0.26 (0.81)	32.18	94965	10.5
2000	14	31.8 (99.22)	0.25 (0.78)	32.02	91312	15.3
2001	21	31.7 (99.16)	0.27 (0.84)	31.98	98618	21.3
2002	20	31.2 (99.17)	0.26 (0.83)	31.45	94965	21.1
2003	19	31.1 (98.95)	0.33 (1.05)	31.40	120532	15.8
2004	8	30.8 (98.94)	0.33 (1.06)	31.09	120532	6.6
2005	21	30.7 (98.72)	0.40 (1.28)	31.14	146100	14.4
2006	19	31.4 (95.52)	0.47 (1.48)	31.86	171668	11.1

1. Traffic flows (total vehicle kilometres divided by the road length of the network in kilometres) are calculated by the Department for Transport and were obtained from the London Travel Report 2007 (7); they are limited to major roads in London.
2. The average annual decrease in death rate was 2.7% per year (95% confidence interval -0.1% to 5.4% per year).
